# Preferential Localization of Fixed Drug Eruption Over a Cosmetic Red Lip Tattoo

**DOI:** 10.7759/cureus.110298

**Published:** 2026-06-05

**Authors:** Yasir Radhi, Ali Almamoori

**Affiliations:** 1 Department of Dermatology, Al-Shaheed Al-Sadr General Hospital, Baghdad, IRQ; 2 Department of Dermatology, Baghdad Teaching Hospital, Baghdad, IRQ

**Keywords:** cosmetic tattooing, drug hypersensitivity, fixed drug eruption (fde), hyperpigmentation, immunocompromised cutaneous district, lip blushing, locus minoris resistentiae, tattoo reaction

## Abstract

Fixed drug eruption (FDE) is a delayed hypersensitivity reaction characterized by recurrent lesions at the same site following re-exposure to an offending medication. Preferential localization over tattooed skin is rare and may mimic tattoo hypersensitivity. We report a 27-year-old woman with recurrent burning, swelling, oozing, and progressive hyperpigmentation predominantly affecting cosmetically tattooed (“lip blushing”) lips. Symptoms began approximately one year after tattoo placement and recurred intermittently. The patient reported episodic use of trimethoprim-containing medications, ibuprofen, and ciprofloxacin preceding several flares. During the most recent episode, a solitary hyperpigmented buttock patch developed. Examination revealed violaceous-gray hyperpigmentation involving the tattooed lips and a well-demarcated buttock patch consistent with healed FDE. The clinical course favored FDE preferentially localized to an immunologically altered cutaneous district created by cosmetic tattooing rather than tattoo hypersensitivity. This case highlights cosmetic tattooing as a potential *locus minoris resistentiae* that may predispose to preferential FDE localization and delay diagnosis.

## Introduction

Fixed drug eruption (FDE) is a distinctive cutaneous adverse drug reaction characterized by recurrent lesions occurring at the same anatomical sites after re-exposure to the offending medication, with an estimated prevalence of approximately 1-5% among all cutaneous drug reactions, though the true incidence is likely underreported due to mild or self-resolving episodes. Lesions commonly present as sharply demarcated erythematous-to-violaceous patches and plaques that may become edematous, erosive, or bullous, subsequently healing with residual post-inflammatory hyperpigmentation. Common causative agents include nonsteroidal anti-inflammatory drugs and antimicrobial agents, particularly sulfonamides and fluoroquinolones [1-4].

The concept of *locus minoris resistentiae*, literally "a site of lesser resistance," refers to a cutaneous district previously altered by trauma, infection, inflammation, or other insults, rendering it immunologically distinct from surrounding skin and predisposing it to preferential localization of subsequent cutaneous reactions. This phenomenon has been documented at sites of healed burns, insect bites, herpes zoster, and venipuncture [1,2].

Tattoo-associated inflammatory reactions are well recognized, especially with red pigments, which are considered the most immunologically reactive tattoo color. Reported reactions include allergic, lichenoid, granulomatous, and pseudolymphomatous patterns [5-8]. Cosmetic tattooing, including lip blushing, has grown substantially in prevalence, with surveys estimating approximately 30% of adults in Western populations carry at least one tattoo, with cosmetic facial tattooing representing a rapidly expanding subset. Preferential localization of FDE over a cosmetic lip tattoo, however, has rarely been described.

We report a case of recurrent, presumptive FDE involving a cosmetic red lip tattoo, initially mimicking tattoo hypersensitivity, with the subsequent appearance of a distant lesion raising the clinical possibility of systemic drug-induced disease.

## Case presentation

A 27-year-old woman presented with progressive dark pigmentation involving the lips following recurrent episodes of swelling, burning sensation, and oozing. Three years earlier, she had undergone cosmetic red lip tattooing (“lip blushing”) involving the entire upper and lower vermilion lips for cosmetic enhancement in a salon setting. Approximately one year after tattoo placement, she began developing intermittent inflammatory episodes localized predominantly to the tattooed lip area, characterized by burning sensation, edema, erythema, and occasional oozing, followed by persistent hyperpigmentation after resolution. Initially, the patient did not associate the episodes with medication exposure.

Structured medication history, obtained using descriptive strategies (pill color, packaging, and generic drug names), revealed intermittent use of trimethoprim-containing medications, ibuprofen, and ciprofloxacin preceding several flares. Importantly, the patient herself independently recognized a pattern of episodes following medication intake, particularly in the context of recurrent urinary tract infection treatment, though she could not reliably identify the specific medication preceding each individual flare. The patient reported that inflammatory episodes developed within approximately six hours following medication use, consistent with the expected temporal pattern of FDE recurrence (typically 30 minutes to six hours).

**Figure 1:**
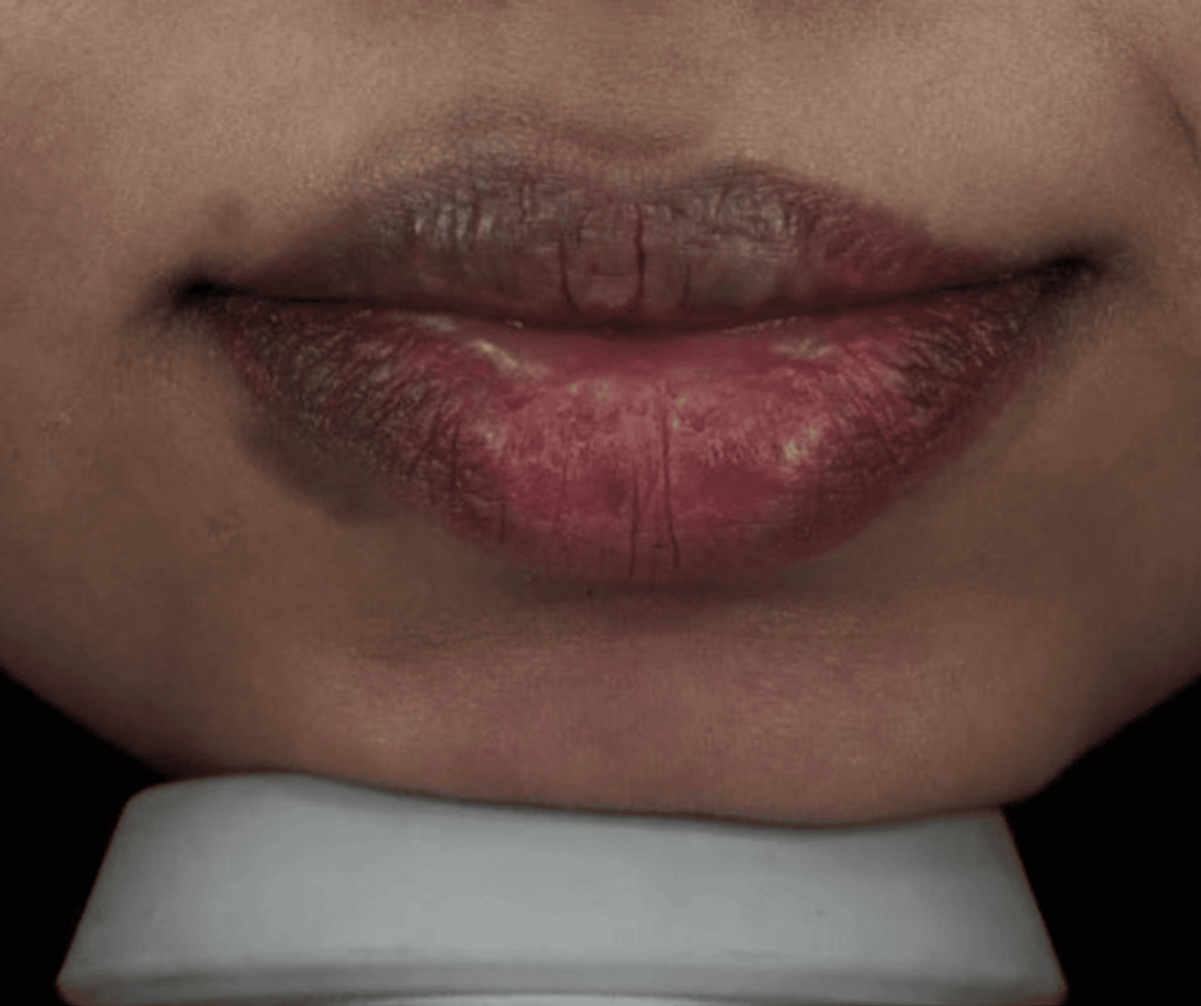
Violaceous-to-slate gray hyperpigmentation involving both upper and lower lips with residual erythematous foci corresponding to prior cosmetic lip tattooing (“lip blushing”). The pigmentation extends slightly beyond the vermilion border onto the adjacent perioral skin.

During the most recent episode, a solitary, round, hyperpigmented patch developed on the buttock. Cutaneous examination demonstrated violaceous-to-slate gray hyperpigmentation involving both upper and lower lips, with residual erythematous areas corresponding to prior cosmetic tattooing (Figure 1).

**Figure 2:**
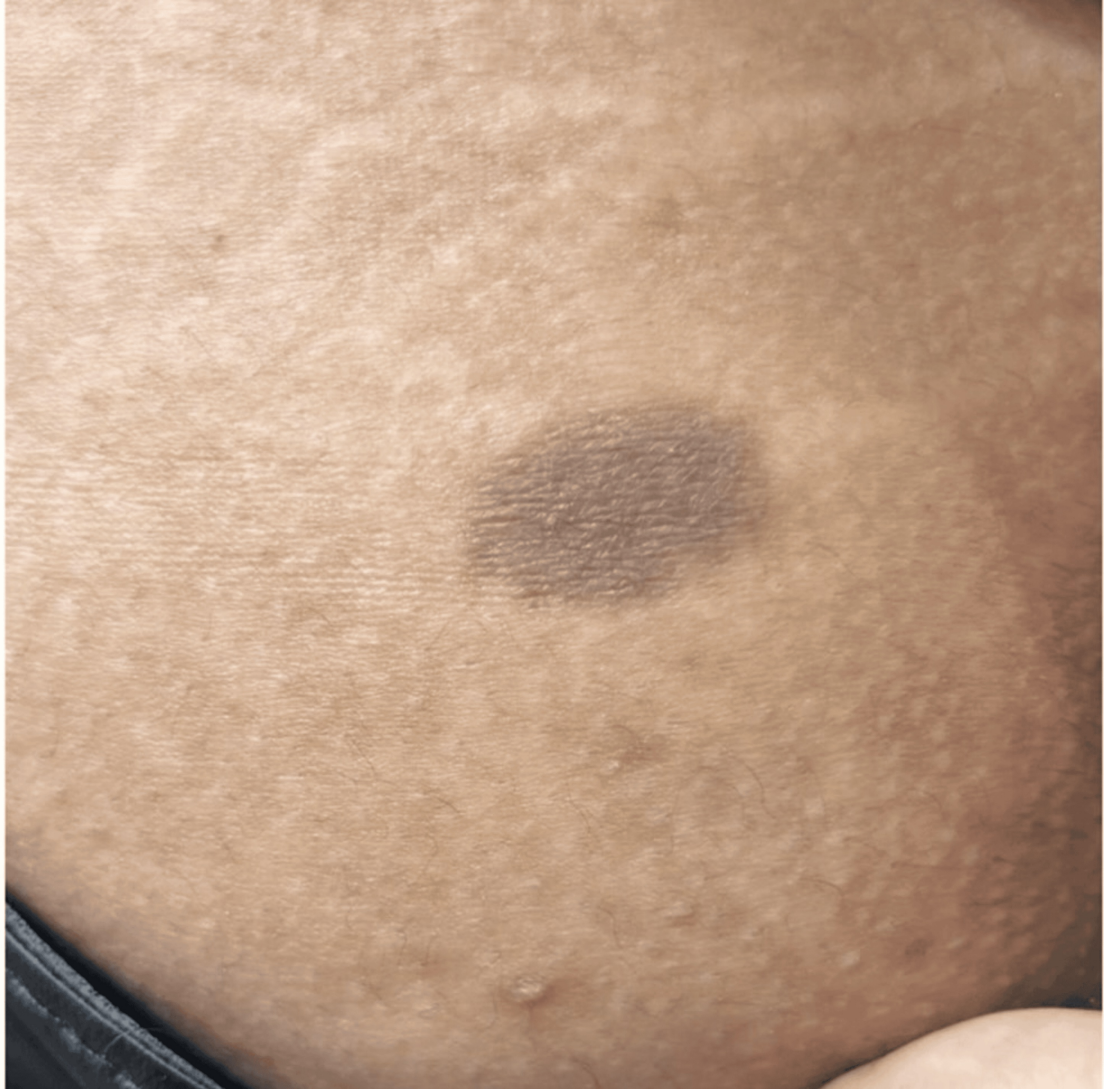
Solitary, well-circumscribed, round hyperpigmented patch on the buttock, consistent with a resolved fixed drug eruption.

The hyperpigmentation involved nearly the entire upper and lower vermilion lips bilaterally, with extension approximately 3-5 mm beyond the vermilion border onto the adjacent perioral skin. A solitary well-demarcated round hyperpigmented patch was observed on the buttock, measuring approximately 2 × 1.8 cm, with uniform hyperpigmentation and no surface change or induration (Figure 2).

Routine blood work was performed at the time of presentation, approximately one month after resolution of the acute episode. All values were within normal limits, reflecting the post-inflammatory quiescent phase. Inflammatory markers, eosinophil counts, and liver enzymes would have been considerably more informative if obtained during an active flare. Immunoglobulin levels and formal immunologic panels were not performed, as there were no clinical features suggesting systemic immunologic disease. The Naranjo adverse drug reaction probability scale was applied (Table 1) [9].

**Table 1:** Naranjo adverse drug reaction probability assessment. The Naranjo adverse drug reaction (ADR) probability scale was applied [9]. Transparent scoring, including its limitations, is provided. A score of four places this case in the “possible” category (scores 5-8 = probable; ≥9 = definite), accurately reflecting the level of diagnostic certainty.

Criterion	Score
Previous conclusive reports on this reaction	+1
Adverse event appeared after the suspected drug was administered	+2
Adverse event improved upon drug withdrawal	+1
Adverse event reappeared on re-administration (formal re-challenge not performed)	0
Alternative causes (tattoo hypersensitivity cannot be excluded)	−1
Reaction appeared when a placebo was given (not done)	0
Drug detected in toxic concentration (not done)	0
Reaction severity varied with dose (not assessed)	0
Similar reaction to the same/similar drugs previously	+1
Adverse event confirmed by objective evidence (no biopsy or patch test)	0
Total Score - Possible ADR (scores 1-4)	4

Based on the clinical picture, a presumptive diagnosis of FDE preferentially localized to the tattooed lip area was considered. The patient was advised to avoid the suspected medications and was counseled regarding the probable drug-related nature of the condition. Trimethoprim-containing medications, especially co-trimoxazole (trimethoprim-sulfamethoxazole), are considered the most probable trigger, given their well-established association with FDE, the patient’s frequent use during recurrent urinary tract infection episodes, and the temporal clinical pattern.

## Discussion

FDE is a delayed type IV hypersensitivity reaction mediated by epidermal CD8+ resident memory T cells. Re-exposure to the offending medication triggers localized inflammation at previously sensitized sites, producing recurrent erythematous, edematous, erosive, or bullous lesions that characteristically resolve with residual post-inflammatory hyperpigmentation [3,4]. Common causative agents include sulfonamides, tetracyclines, nonsteroidal anti-inflammatory drugs, and fluoroquinolones.

Tattoo-associated inflammatory reactions are more frequently observed with red pigments and may present as allergic contact dermatitis, lichenoid reactions, granulomatous inflammation, or pseudolymphomatous reactions [5-8]. These reactions are typically confined to tattooed skin and do not produce distant lesions, which are more suggestive of a systemic drug-induced process.

In the present case, the initial preferential involvement of a cosmetically tattooed red lip area created significant diagnostic overlap between FDE and tattoo hypersensitivity. However, two findings argue against a purely localized tattoo reaction: extension of hyperpigmentation approximately 3-5 mm beyond the tattoo margins onto adjacent perioral skin and the development of a distant, well-demarcated hyperpigmented patch on the buttock. The latter lesion was not biopsied and therefore cannot be considered confirmatory; both findings are interpreted as clinical observations suggestive of systemic drug involvement rather than definitive evidence. We acknowledge the limitation that correlating unconfirmed lesions may introduce circular reasoning.

One possible explanation is that the tattooed vermilion lip acted as an immunocompromised cutaneous district or *locus minoris resistentiae*. Repeated mechanical trauma from cosmetic tattooing, chronic low-grade inflammation, and persistent pigment deposition may alter local immune regulation, predisposing the site to preferential localization of drug-induced inflammation [10,11]. This hypothesis remains speculative, and no direct histopathologic or immunologic confirmation is available in this patient. The process may also be interpreted within a Koebner-like or isotopic response spectrum, although caution is warranted in the absence of biopsy-proven disease.

Trauma-localized FDE has previously been reported at sites of healed burns, insect bites, herpes zoster, and venipuncture sites, supporting the concept of preferential disease localization in previously altered skin [1,2]. To our knowledge, preferential localization of presumptive FDE over a cosmetic lip tattoo with a subsequent distant lesion has not been previously reported.

From a management perspective, immediate withdrawal of the suspected offending drug remains the cornerstone of therapy. The patient was counseled to avoid trimethoprim-containing medications, nonsteroidal anti-inflammatory drugs, and fluoroquinolones, which are recognized triggers of FDE. During acute episodes, topical corticosteroids and oral antihistamines may provide symptomatic relief, while short courses of systemic corticosteroids can be considered in more extensive disease.

Residual post-inflammatory hyperpigmentation, particularly over tattooed skin, is difficult to treat. Options include topical depigmenting agents such as hydroquinone, azelaic acid, kojic acid, and tranexamic acid combined with strict photoprotection. Laser therapies (e.g., Q-switched Nd:YAG) may be considered with caution due to potential alteration of tattoo pigment and unpredictable pigmentary outcomes. Preventing recurrence through strict drug avoidance remains the most effective long-term strategy, as repeated episodes can exacerbate pigmentary changes.

In cases of future recurrence, diagnostic confirmation would ideally include punch biopsy during the active phase (within 48-72 hours), laboratory evaluation during flare, and patch testing with suspected drugs at least six weeks after resolution. Prospective medication documentation and standardized photographic monitoring are also recommended to strengthen temporal correlation and improve diagnostic certainty.

Overall, this case highlights cosmetic lip tattooing as a potential immunologically altered cutaneous site that may predispose to preferential localization of FDE, thereby complicating diagnosis and delaying recognition of a systemic drug-induced eruption.

## Conclusions

This case highlights the possibility that cosmetic lip tattooing may act as an immunocompromised cutaneous district (*locus minoris resistentiae*), predisposing to preferential localization of FDE at the tattooed site. The recurrent inflammatory episodes, persistent post-inflammatory hyperpigmentation extending beyond tattoo margins, and development of a distant lesion support a systemic drug-induced process rather than an isolated tattoo hypersensitivity reaction, although histopathologic confirmation was unavailable. FDE should be considered in patients presenting with recurrent reactions at cosmetic tattoo sites, especially following medication exposure. Diagnostic evaluation should include causality assessment (e.g., Naranjo scale), biopsy during active lesions, patch testing when available, and careful medication review. As cosmetic tattooing becomes increasingly common worldwide, awareness of this uncommon presentation is important to facilitate timely diagnosis, avoid recurrent drug exposure, and encourage reporting of similar cases to strengthen the evidence base.
